# Matrix Stiffness Enhances Odontogenic Differentiation of DPSCs through Membrane Curvature Protein Baiap2‐Modulated Exosome Release

**DOI:** 10.1002/advs.76908

**Published:** 2026-08-11

**Authors:** Bilun Jin, Shaojie Dong, Yue Jia, Zhao Xu, Peiqi Liu, Zhaojing Ding, Yuxin Liao, Rui Zou, Jinsong Hu, Bo Cheng, Lin Niu

**Affiliations:** ^1^ Key Laboratory of Shaanxi Province For Craniofacial Precision Medicine Research, College of Stomatology Xi'an Jiaotong University Xi'an China; ^2^ Clinical Research Center of Shaanxi Province For Dental and Maxillofacial Diseases, College of Stomatology Xi'an Jiaotong University Xi'an China; ^3^ College of Stomatology Xi'an Jiaotong University Xi'an China; ^4^ School of Life Science and Technology The Key Laboratory of Biomedical Information Engineering of the Ministry of Education Xi'an Jiaotong University Xi'an China; ^5^ Bioinspired Engineering and Biomechanics Center (BEBC) Xi'an Jiaotong University Xi'an China; ^6^ Department of Cell Biology and Genetics School of Basic Medical Sciences Xi'an Jiaotong University Xi'an Shaanxi China

**Keywords:** Baiap2, dental caries, exosome, matrix stiffness, membrane curvature

## Abstract

Altered extracellular biomechanical forces are increasingly implicated in a wide range of pathologies. However, the specific role of extracellular matrix stiffness in the pathogenesis of dental caries remains unclear. Although exosomes have been shown to regulate cell fate, it is still poorly understood how their biological journeys from biogenesis to secretion are affected by matrix stiffness. Here we find that matrix stiffness can enhance exosome release, thereby promoting odontogenic differentiation of dental pulp stem cells (DPSCs). Mechanistically, matrix stiffness promotes exosome release by modulating membrane curvature through Baiap2, which is tightly regulated by the stiffness‐activated PI3K‐AKT signaling pathway. Moreover, the release of exosomes was found to depend on mechanosensitive motor protein kinesin‐1 and its regulation of vesicular transport. Taken together, our results demonstrate that matrix stiffness plays an essential role in odontogenic differentiation of DPSCs by modulating exosome release. Our findings prove that the mechanosensitive process may play an underappreciated role in regulating exosome secretion and odontogenic differentiation in deep caries.

## Graphical abstract

Matrix stiffness softening is identified as a previously underappreciated feature in deep caries. Higher stiffness drives exosome release in dental pulp stem cells through a coordinated mechanism involving PI3K–AKT‐regulated Baiap2 and kinesin‐1, thereby promoting odontogenic differentiation. The discovery of this mechanosensitive process deepens the understanding of pathogenesis in deep caries.

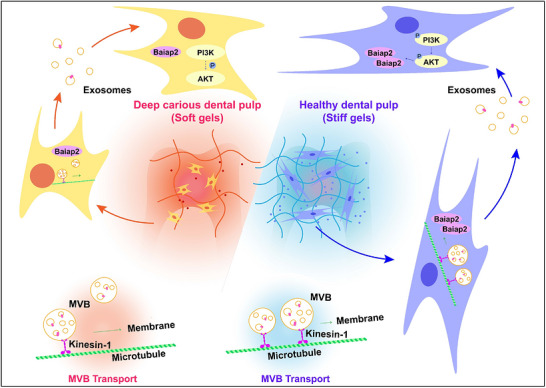

## 1 Introduction

Dental caries remains one of the most prevalent oral diseases worldwide, representing a significant public health challenge [1]. In chronic dentinal caries lesions, a heightened expression of cathepsin proteinases in odontoblasts and dentinal tubules, together with matrix metalloproteinases (MMPs) in saliva would lead to extracellular matrix (ECM) degradation and tissue resorption [2]. This pathological remodeling may alter the mechanical properties of dental pulp. Matrix stiffness is one of the most important mechanical cues in regulating cellular behaviors [3]. Recent advances in regenerative endodontics have highlighted the therapeutic potential of dental pulp stem cells (DPSCs), owing to their ability to promote pulp‐dentin regeneration as a promising approach to treat dental caries [4]. Emerging evidence suggests that matrix stiffness is a critical determinant of the fate of DPSCs, affecting cell proliferation, differentiation and migration [5, 6, 7, 8]. These findings indicate the importance of mechanosensitive signaling pathways in the pathophysiological process of dental caries. However, the matrix stiffness of healthy and diseased pulp, particularly in the context of deep caries, remains largely unexplored, thereby limiting the development of stiffness‐targeted interventions.

Exosomes have emerged as critical mediators of intercellular communication, and are involved in various physiological and pathological processes. Nearly all cells are capable of producing exosomes, and the biogenesis of exosomes is a complex and highly regulated process: from the initial generation of early endosomes, formation and maturation of multivesicular bodies (MVBs), to the final fusion between MVBs and plasma membrane for mature exosome release into the extracellular environment [9, 10, 11]. Abnormalities in exosome biogenesis, cargo sorting, and release have also been found to be associated with numerous diseases [12, 13, 14]. Moreover, changes in ECM properties, such as viscoelasticity and dimensionality, could significantly affect exosome yield, size and protein cargo [15, 16]. In this regard, as another important biophysical signal of ECM, matrix stiffness has also been shown to control exosome production [17, 18, 19, 20]. However, the mechanisms by which matrix stiffness modulates exosomes remain poorly understood. Given the stiffness changes of ECM in dental caries, it is crucial to investigate the relationship between matrix stiffness and exosomes, as well as the role of this interaction in deep caries.

In this study, we validated matrix softness as a defining pathological feature of deep carious pulp tissue by directly measuring the matrix stiffness of fresh human dental pulp samples using a rheometer. Employing hydrogels with different stiffness, we further investigated how matrix stiffness affects the odontogenic differentiation of DPSCs through modulating exosome release. This process involves intracellular vesicle transport and release driven by kinesin‐1 and PI3K‐AKT‐regulated‐Baiap2. Our findings reveal an extracellular matrix stiffness‐dependent regulation mechanism of exosome secretion in the pathological process of deep caries, providing new insights into the mechanobiology of this disease.

## 2 Materials and Methods

### 2.1 Isolation, Culture, and Identification of hDPSCs and Carious hDPSCs

All experiments were approved by the Ethics Committee of Xi'an Jiaotong University (KY‐GXB‐20230002). Written informed consent was obtained from all donors prior to sample collection. Extracted teeth were collected from donors aged 18 to 25 years. Normal pulp tissues were collected from freshly extracted third molars without any diseases. Carious pulp tissues were obtained from third molars diagnosed with coronal deep caries. The diagnosis of deep caries was based on the assessment of two endodontic specialists. Teeth with carious lesions extending beyond inner one‐third of dentin thickness and a remaining dentin thickness of ≤ 2 mm, but without irreversible pulpitis were classified as deep carious teeth [21, 22]. These lesions were considered as chronic caries based on their clinical and radiographic presentation.

After extraction, teeth were immediately placed in pre‐cooled PBS solution containing 10% penicillin/streptomycin antibiotics (Gibco, USA) and transferred to the laboratory. The coronal dental pulp tissues were extracted and placed into a 10 cm dish. All pulp tissues were minced into fragments with a diameter ≤ 0.5 mm and digested with 3 mg/mL collagenase type I (Gibco, USA) for 30 min at 37°C to obtain the dental pulp tissue suspension. The suspension was centrifuged at 1000 r/min for 5 min and the sediments were resuspended in α‐Minimal Essential Medium (α‐MEM, Gibco, USA), containing 10% fetal bovine serum (FBS, TIANHANG, China) and 1% penicillin/streptomycin (Biosharp, China). Then the suspension was added in a culture dish and incubated in a 5% CO_2_ atmosphere at 37°C for 5 days. Once the cells migrated out, the media was changed every other day until the cell confluence reached 80%. Cells were then detached from the dish with a 0.25% trypsin‐EDTA (Gibco, USA) and passaged every 3 days.

The characteristics of hDPSCs and deep carious hDPSCs (ChDPSCs) were identified by the following assays including multi‐differentiation assays, flow cytometric analysis of stem cell‐specific markers CD45, CD73, CD90 and CD105. Table S1 provides a list of antibodies used for flow cytometry. For all experiments, DPSCs between passages 4 and 6 were employed.

### 2.2 EdU Cell Proliferation Assays

Cell proliferation was analyzed using an EdU kit (Beyotime, China). Briefly, 1×10^5^ cells/well seeded into 6‐well plates were incubated with 10 µM EdU for 12 h. Then, cells were fixed with 1 mL of 4% paraformaldehyde for 30 min, and permeabilized with 0.3% Triton‐X100 for 10 min. After incubating with Click reaction solution in the dark for 30 min, and counterstaining nuclei with Hoechst for 10 min, the cells were observed by an Axioplan 2 fluorescence microscope (Carl Zeiss, Germany).

### 2.3 ALP Assay

Following 7 days of osteogenic induction, DPSCs were fixed with 4% paraformaldehyde for 30 min at room temperature and washed three times with PBS. The cells were then incubated with 1 mL alkaline phosphatase (ALP) staining solution at 37 °C for 1 h. The results were imaged under an Axioplan 2 fluorescence microscope (Carl Zeiss, Germany).

Semiquantitative analyses of ALP were performed based on the protocol provided in the ALP detection kit (Beyotime, China). Briefly, cells were washed by PBS for 3 times and then treated on ice for 30 min with RIPA buffer (Boster, China) without phosphatase inhibitors. Lysates were collected and centrifuged at 12,000 r/min for 20 min at 4°C. 25 µL of each supernatant was then seeded in a well of one 96‐well plate and incubated with 50 µL of *p*‐Nitrophenyl phosphate and 25 µL of the detection buffer at 37°C for 10 min. Then the optical density (OD) values of reaction product in each well were measured at 405 nm to compare the ALP activity.

### 2.4 Alizarin Red S Staining (ARS)

After osteogenic induction for 21 days, DPSCs were fixed with 4% paraformaldehyde for 30 min. After washing with PBS, DPSCs were stained with 1 mL ARS solution for 10 min. Then the stained cells were visualized via an Axioplan 2 fluorescence microscope (Carl Zeiss, Germany). ARS quantification was performed by adding 10% cetylpyridinium chloride (Macklin, China) to each well, followed by OD measurement at 560 nm.

### 2.5 Oil Red O Staining

After adipogenic induction for 21 days, DPSCs were washed with PBS, fixed with 4% paraformaldehyde for 30 min, and stained with Oil Red O staining solution for 30 min. Images were captured using an Axioplan 2 fluorescence microscope (Carl Zeiss, Germany).

### 2.6 Mechanical Characterization of Deep Carious Dental Pulps

Fresh tissues and hydrogels were prepared at a thickness of 1 mm and a diameter of 4 mm, and rheology tests were carried out using an MCR 302 rheometer (Anton Paar, Austria). Frequency sweep measurements were performed at a constant strain of 1%, and the storage modulus (G′) at 6.28 rad/s was recorded. All tests were performed using more than three independent biological replicates. The mean of these measurements was used to represent the final modulus values.

### 2.7 Bioinformatics

Single cell RNA sequencing data were obtained from the GEO databases (GSE185222, https://www.ncbi.nlm.nih.gov/geo/query/acc.cgi?acc = GSE185222). Genes with low expression levels were removed by ‘Seurat’ package applied in R software (version 4.2.2) [23]. After data standardization and normalization, dimensionality reduction via Principal Component Analysis (PCA), spatial organization was performed using t‐Distributed Stochastic Neighbor Embedding (t‐SNE). Then cells with stem cell markers were recognized as DPSCs to filter out key genes or pathways related to DPSCs in deep carious dental pulp.

### 2.8 Hematoxylin and Eosin (H&E) Staining

Dental pulp tissues were first fixed with formalin and embedded in paraffin blocks. Following deparaffinization, all sections were rehydrated and subsequently stained with hematoxylin and eosin. Images were captured under a bright field microscope (Carl Zeiss, Germany).

### 2.9 Immunohistochemistry (IHC)

Dental pulp tissues were fixed with formalin and embedded in paraffin blocks. All sections were dewaxed with xylene, rehydrated with gradient ethanol, followed by being treated in citrate buffer with pH 6.0 for antigen retrieval. Endogenous peroxidase was blocked with 3% H_2_O_2_ in methanol for 30 min. Nonspecific binding was then blocked with 2% bovine serum albumin for 20 min at room temperature. The sections were incubated with Rabbit anti‐ITGB1 (Proteintech 12594‐1‐AP, 1:200) overnight at 4°C. Sections were then incubated with HRP‐secondary antibody for 50 min at room temperature. After staining with DAB, sections were counterstained with hematoxylin. Images were captured under a bright field microscope (Carl Zeiss, Germany).

### 2.10 Flow Cytometry

DPSCs were trypsinized, washed and resuspended in 100 µL PBS to a concentration of 2 × 10^6^ cells/mL. The cells were then stained with fluorochrome‐conjugated antibodies (5 µL/test) against CD45, CD73, CD90, CD105 and ITGB1 for 30 min at 4°C. After being washed twice with PBS, samples were analyzed with a flow cytometer (CytoFLEX SRT) (Beckman, USA). The flow cytometry data were analyzed using CytExpert SRT software (Beckman, USA). The antibodies used for flow cytometry were listed in Table S1.

### 2.11 Western Blotting

The protein samples were obtained with RIPA buffer (Boster, China), and the protein concentration was quantified with a BCA assay kit, then denatured in a boiling bath at 95°C for 10 min. Next, equal amounts of proteins were separated by the 10% sodium dodecyl sulfate‐polyacrylamide gel electrophoresis (SDS‐PAGE) and transferred to PVDF membranes (Boster, China). The PVDF membranes were washed with blocking buffer (Seven, China) for 30 min, and then incubated with primary antibodies at 4°C overnight. The membranes were then incubated with HRP‐conjugated secondary antibody (Boster, China) at room temperature (1 h). The protein bands were visualized by ECL Western Blotting Substrate (Boster, China) and a Chemidoc MP Imaging system (BioRad, USA). The primary antibodies and dilutions used for western blotting were listed in Table S2.

### 2.12 Preparation and Mechanical Characterization of Polyacrylamide (PA) Gels

Polyacrylamide (PA) gels were fabricated by combining specific amounts of acrylamide (Acr) and bisacrylamide (Bis). Stock solutions were prepared as follows: 50% Acr was made by dissolving 10 g of Acr powder (Macklin, China) in deionized water to a final volume of 20 mL, 1.25% Bis stock was made by dissolving 0.5 g of Bis powder (Macklin, China) in deionized water to a final volume of 40 mL, 10% APS was prepared by dissolving 0.5 g of APS powder (Macklin, China) in 5 mL of deionized water. All stock solutions were stored at 4°C protected from light, except APS which was stored at ‐20°C. The compositions of PA hydrogels were shown in Table S3.

For swelling tests, 300 µL of the polymerization solution was pipetted into a sterile 15 cm bacterial culture dish, and a 30 mm round hydrophilic glass coverslip was immediately placed over the droplet. After polymerization at room temperature for 30 min, each gel was transferred to a 6‐well plate containing 2 mL PBS in each well. The swelling ratio of hydrogels was calculated at designated time points as (𝑊_𝑡_−𝑊_0_)/𝑊_0_×100%, where 𝑊_𝑡_ and 𝑊_0_ were the swollen and initial dry weights, respectively.

For rheological measurements, gels were prepared using a western blot gel casting stand, punched into 1 cm diameter disks using a biopsy punch, and immersed in PBS for 2 days to reach swelling equilibrium. They were maintained in PBS until the designated time points. Measurements were performed using a rheometer (Anton Paar, Austria) at 37°C. Frequency sweep measurements were performed at a constant strain of 1%, and the storage modulus (G′) at 6.28 rad/s was recorded. Additionally, atomic force microscopy (AFM) measurements were performed using a silicon nitride cantilever with a spherical silica bead at its tip (spring constant 0.06 N/m). Force‐distance curves were acquired in force spectroscopy mode in PBS at room temperature, with a trigger threshold of 1.5 nN and a feedback setpoint of 0.8 V. For each sample, 4 force maps were collected, with 3 independent samples per group, and the Young's modulus was recorded at day 1 and day 7 post‐swelling equilibrium in confocal dishes.

For cell culture, the gels were functionalized with 200 µL Sulfo‐SANPAH (Proteochem, USA) at 0.4 mg/mL. Subsequently, the Sulfo‐SANPAH was crosslinked using ultraviolet (UV) light at 254 nm (rated power 8 W) for 15 min, and this step was repeated twice. Each gel was then coated with 1 mL rat tail collagen type I (Corning, USA) solution at 0.075 mg/mL overnight at 4°C to obtain uniform surface coating. Before each cell experiment, the gels were exposed to UV light at 254 nm (rated power 21 W) for 2 h to ensure sterility, with an estimated fluence of 0.29–0.72 J/cm^2^.

### 2.13 Immunofluorescence Staining

DPSCs cultured on the hydrogels were fixed with 4% paraformaldehyde for 30 min. After being permeabilized with 0.2% Triton X‐100 (Beyotime, China) and treated with the blocking buffer (Boster, China) at room temperature for 10 min respectively, samples were incubated with primary antibodies overnight at 4°C and then labeled with secondary antibody (Boster, China, 1:100). The primary antibodies and dilutions used for immunofluorescence staining were listed in Table S4. Actin‐Tracker Red (Beyotime C2203S, 1:100), Tubulin‐Tracker Red (Beyotime C1050, 1:100) and DAPI (Solarbio C0060, 1:100) were then used to stain samples. Finally, after being washed by PBS for three times, samples were observed and photographed by a confocal microscope (Olympus, Japan).

### 2.14 Isolation of Exosomes

Conditioned cell culture medium from soft and stiff PA gels was collected for isolation of exosomes. 4 × 10^5^ DPSCs were cultured on soft or stiff PA gels (previously functionalized with Sulfo‐SANPAH and coated with Type‐I collagen) in 6‐well plates with serum‐free media (Umibio, China) for one day. The conditioned medium was then collected and centrifuged at 3000 × g for 15 min and filtered through a 0.22 µm filter unit (Millipore, USA) to remove cellular debris and larger vesicles. The supernatant was collected and centrifuged at 120,000 × g for 70 min at 4°C. This washing step was repeated once more. Finally, exosomes were then resuspended in pre‐chilled PBS and stored at ‐80°C for subsequent analysis. The method was widely used and has been demonstrated to yield exosomes with high purity [24, 25].

### 2.15 Characterization of Exosomes

The morphology of exosomes was observed and analyzed by cryogenic electron microscopy (cryo‐EM). Nanoparticle Tracking Analysis (NTA) was used to determine the exosome diameter and particle size distribution. We also used western blotting to validate the exosome markers, including TSG101, CD63, HSP70 and Calreticulin. The primary antibodies used for western blotting were listed in Table S2.

### 2.16 Proteomic Assay

Quantitative proteomic analysis of exosomes from hDPSCs cultured on soft and stiff gels was performed using a Data‐independent acquisition (DIA) LC‐MS/MS platform (Thermos, USA, QE HF‐X) to characterize their protein profiles. The DIA method consisted of one full MS‐SIM scan, followed by 30 DIA scans covering a mass range of 400–1200 m/z per cycle. Finally, bioinformatics analyses were performed with R software (version 4.2.2) to identify proteins with significant expression differences between exosomes derived from hDPSCs cultured on soft and stiff gels.

### 2.17 Quantification of Membrane Curvatures

DPSCs on soft or stiff gels were labeled with F‐actin by immunofluorescence staining and then subsequently imaged using a confocal microscope (Olympus, Japan). The average membrane curvatures of each cell were assessed via the ImageJ plugin Kappa (version 2.0.0), which allowed the user to trace the cell shape in a few clicks and fit that curve with a minimization algorithm [26].

### 2.18 Gene Knockdown and Overexpression

Baiap2‐knockdown cell lines were constructed by transducing lentiviral particles encoding *BAIAP2*‐targeting shRNAs at a multiplicity of infection (MOI) of 35 (Shanghai Genechem Co., Ltd.). An empty lentiviral vector was used as a control. DPSCs were plated onto 6‐well plates, grown to 20%‐30% confluence, and incubated for 12 h after the addition of lentivirus. After 48 h, the cells were incubated in the culture medium containing 2.5 µg/mL puromycin for 7 days to select positive clones, and verified by western blot analysis.

For Baiap2 overexpression, the full‐length human *BAIAP2* coding sequence was cloned into a lentiviral overexpression vector (Beijing Tsingke Biotechnology Co., Ltd.), and lentiviral particles were transduced at an MOI of 35. An empty vector served as the negative control. Following the same protocol as the knockdown method, cells were selected using puromycin and positive clones were confirmed by western blot analysis.

### 2.19 Exosome Internalization

Green fluorescent EvLINK 505 (illuTINGO, China) was used to label exosomes. Briefly, exosomes were incubated with 5 µL EvLINK 505 per 100 µg for 30 min. Then the labeled exosomes were ultrafiltered at 7000 × g for 15 min four times using 100 kDa centrifuge tubes (Millipore, USA). Next, the exosomes were incubated with hDPSCs for 24 h. Finally, the treated hDPSCs were fixed with 4% paraformaldehyde for 30 min. The nuclei and cytoskeleton were stained with DAPI (Solarbio, 1:100) and Actin‐Tracker Red (Beyotime, 1:100) after being washed with PBS for three times. Exosomal internalization was observed and photographed by a confocal microscope (Olympus, Japan).

### 2.20 Pharmacological Inhibition of Kinesin‐1

Rose Bengal lactone (RBL) was dissolved in DMSO to prepare a 40 mM stock solution. 2 µL of the stock solution was added to 2 mL of culture medium per well to achieve a final concentration of 40 µM. The stock solution was stored at ‐20°C and protected from light. The concentration of RBL was selected based on previous literature and preliminary optimization [27].

### 2.21 Mathematical Model

A mathematical model was developed to simulate the relationship between membrane tension and exosome release under the regulation of different matrix stiffness. This was achieved by expanding a chemical model described elsewhere [28].

### 2.22 Statistical Analysis

All tests were performed more than three times independently. For Western blot band density quantification and exosome release comparisons, the raw data were normalized to the mean of the control group before statistical analysis. Most data were presented as mean ± standard deviation (SD), while the distribution data of molecules in the transport system were presented as mean ± standard error of the mean (SEM). Comparisons were performed by Student's two‐tailed *t*‐test for two groups and one‐way ANOVA followed by Tukey's post hoc test for multiple groups. *P* < 0.05 was considered statistically significant. The sample size for each experiment was described in the corresponding figure legends. All data were analyzed by GraphPad Prism 8.0.

## 3 Results

### 3.1 Soft Matrix Stiffness is Associated with the Decreased Mineralization and Exosome Secretion in Deep Carious DPSCs

**FIGURE 1:**
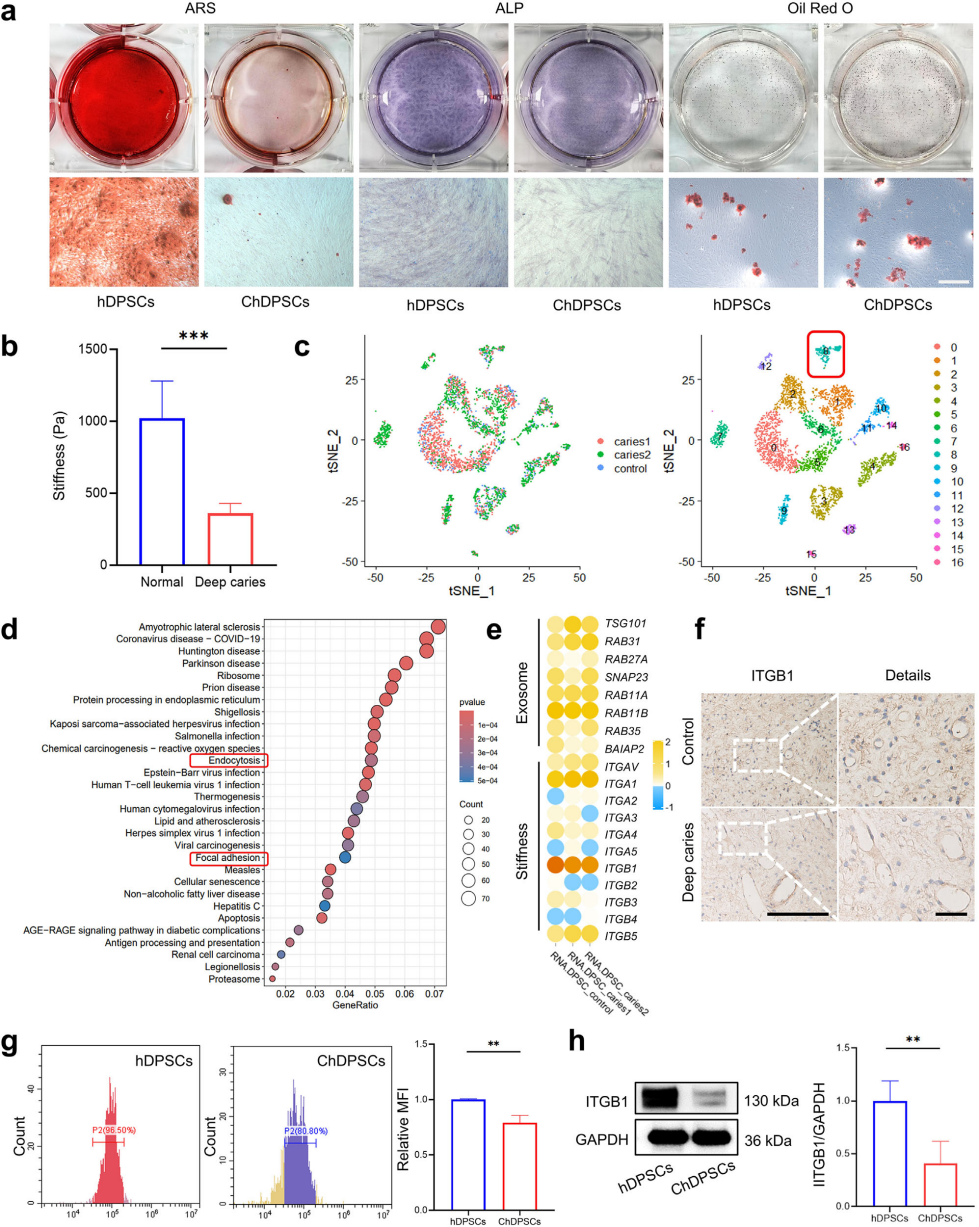
The decreased mineralization ability of carious DPSCs is associated with the variance of matrix stiffness and exosome secretion. (a) The comparation of adipogenic and osteogenic differentiation of hDPSCs and ChDPSCs (*n* = 3). Scale bar = 100 µm. (b) The stiffness of healthy and deep carious dental pulp tissues (*n* = 5). (c) The t‐SNE visualization of single‐cell RNA‐seq data. Each cluster was coded with different color to identify the subtype cells. (d) The diagram of 10 most enriched KEGG terms. (e) Heatmap of genes associated with stiffness and exosomes identified in hDPSCs and ChDPSCs. (f) Representative immunohistochemical staining images of ITGB1 in healthy and deep carious dental pulp tissues. Scale bar = 100 µm. (g) The MFI of ITGB1 in hDPSCs and ChDPSCs examined by flow cytometry (*n* = 3). (h) Western blotting analysis of the expression of ITGB1 in hDPSCs and ChDPSCs (*n* = 4). Data are expressed as mean ± SD. Statistical analysis was performed using Student's two‐tailed *t*‐test. *p < 0.05, **p < 0.01, ***p < 0.001.

Given the potential impact of deep caries on DPSCs, we initially analyzed stem cell marker expression and compared the differentiation profiles of hDPSCs and ChDPSCs. Based on the results of ALP assay, ARS staining, and Oil Red O staining (Figure 1a), both hDPSCs and ChDPSCs possess osteogenic and adipogenic differentiation potential. However, hDPSCs exhibit stronger osteogenic/odontogenic differentiation capabilities than ChDPSCs, while ChDPSCs show superior adipocyte differentiation potential. As shown in Figure S1, hDPSCs and ChDPSCs had similar expression levels of stem cell markers. Both hDPSCs and ChDPSCs exhibited high expression of stem cell surface markers CD73, CD90, and CD105, but negative for the hematopoietic marker CD45. In addition, hDPSCs showed a superior proliferative capacity (Figure S2).

As matrix stiffness is a critical determinant of stem cell differentiation, we next measured the stiffness of healthy and deep carious dental pulp to investigate its potential association with the reduced mineralization capacity of DPSCs [29]. Quantitative comparison confirmed that the matrix stiffness of deep carious pulp (360.44 ± 68.41 Pa) was significantly lower than that of healthy pulp (1022.91 ± 256.76 Pa) (Figure 1b, Figures S3 and S4, Table S5). These results indicate a marked difference in tissue stiffness between the two conditions.

To identify the functional characteristics of differentially expressed genes and the potential mechanisms involved in deep carious DPSCs, we analyzed the differential gene expression profiles between hDPSCs and ChDPSCs using single‐cell RNA sequencing data. Subcluster 8 was identified as stem cells based on the expression of positive markers *MCAM (CD146)* and *THY1 (CD90)*, and the absence of the negative marker *PTPRC (CD45)* (Figure 1c and Figure S5a). Subsequent analysis of differentially expressed genes, followed by Kyoto Encyclopedia of Genes and Genomes (KEGG) enrichment using the GEO database (GSE185222), revealed that endocytosis and focal adhesion pathways were associated with the pathological process (Figure 1d). This finding strongly implicated ECM and exosome‐related mechanisms. To further explore this link, we performed a correlation analysis on representative genes related to stiffness and exosomes. *ITGB1* (integrin β1) was highly abundant, with significantly higher levels in the hDPSC group (Figure 1e). Moreover, *ITGB1* expression correlated strongly with multiple exosome‐related genes (Figure S5b), suggesting a potential association between matrix stiffness and exosome biogenesis.

To verify the difference in ITGB1 expression between hDPSCs and ChDPSCs, we performed immunohistochemistry, flow cytometry and western blotting to measure the ITGB1 expression in different samples. Both flow cytometry and western blotting data confirmed that the expression of ITGB1 in hDPSCs was significantly higher than that in ChDPSCs (Figure 1f–h). These findings demonstrate that the matrix hardness of deep carious pulp is reduced compared to that of healthy pulp, strongly suggesting a correlation between matrix stiffness and the impaired odontoblast differentiation capacity of ChDPSCs.

### 3.2 Stiffness Regulates the Odontogenic Differentiation Capacity of hDPSCs and Carious hDPSCs

**FIGURE 2:**
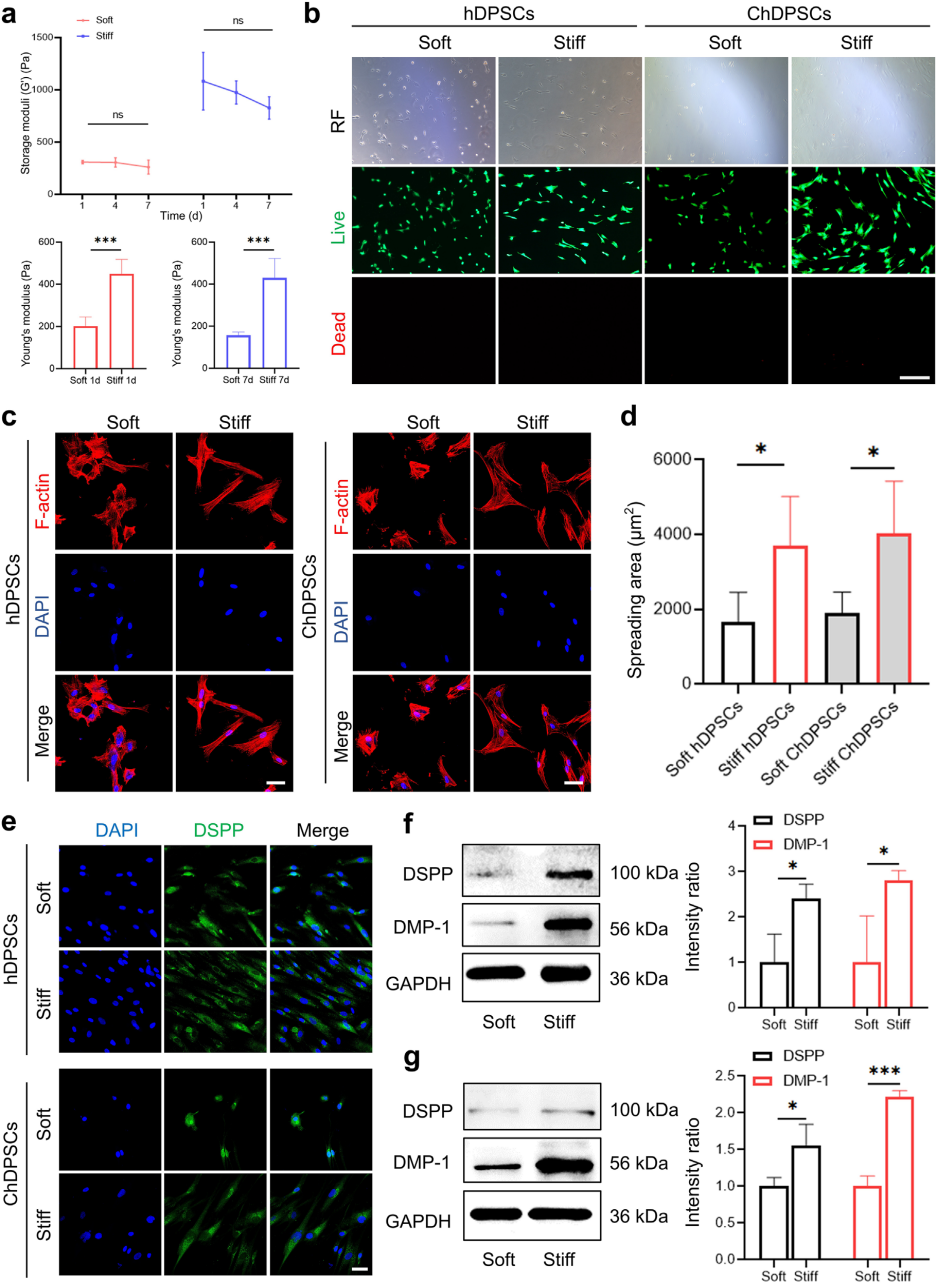
DPSCs on stiffer hydrogels have better odontogenic differentiation ability. (a) The stiffness of soft and stiff hydrogels during seven days (*n* = 3). (b) Representative images of live and dead assays. Scale bar = 100 µm. (c) Influence of matrix stiffness on the morphology of hDPSCs and ChDPSCs. Cells were labeled with actin cytoskeleton (red) and DAPI (blue) for nuclei. Scale bar = 50 µm. (d) The spreading area of cells incubated on gels with different stiffness (*n* ≥ 4). (e) Representative confocal images of cells labeled with DSPP (green) and DAPI (blue) for nuclei. Scale bar = 50 µm. (f) The protein levels of DSPP and DMP‐1 in hDPSCs cultured on soft or stiff gels were analyzed by western blotting (*n* = 3). (g) The protein levels of DSPP and DMP‐1 in ChDPSCs cultured on soft or stiff gels were analyzed by western blotting (*n* = 3). Data are expressed as mean ± SD. Statistical analysis was performed using Student's two‐tailed *t*‐test between two groups and one‐way ANOVA followed by Tukey's post‐hoc test among three groups. *p < 0.05, **p < 0.01, ***p < 0.001, ns not significant.

To prove that matrix stiffness regulates DPSC odontogenic differentiation independently, we investigated the effects of matrix stiffness on hDPSCs and ChDPSCs under non‑inflammatory conditions. We cultured DPSCs on polyacrylamide (PA) hydrogels with approximately 300 Pa (soft) and 1000 Pa (stiff), which fall within the range of our rheological measurements of deep carious and healthy human dental pulp, respectively. Hydrogel swelling experiments showed that both hydrogels essentially reached swelling equilibrium after 48 h (Figure S6). Despite some swelling, the hydrogels exhibited remarkable mechanical stability over the 7‐day culture period. Rheological measurements showed that the stiff hydrogels exhibited storage moduli (G') of 1082.95 ± 275.68 Pa on day 1 and 826.90 ± 106.79 Pa on day 7 post‐swelling equilibrium, and the soft hydrogels showed G' values of 308.88 ± 16.39 Pa and 260.32 ± 67.15 Pa, respectively. No significant differences were observed between day 1 and day 7 for either group, indicating that the hydrogel stiffness maintained stable throughout the 7‐day culture period (Figure 2a). Young's modulus measured by AFM showed that at both day 1 and day 7, the stiff hydrogels exhibited significantly higher Young's modulus than the soft hydrogels, confirming that both hydrogels maintained a stable mechanical contrast throughout the culture period (Figure 2a).

The results of live/dead staining confirmed excellent cell compatibility of both the soft and stiff gels (Figure 2b). We also evaluated the proliferative capacity of both cell types. As shown in Figure S7, hDPSCs maintained a higher proliferation rate than diseased counterparts on both soft gels and stiff gels. Notably, matrix stiffness influenced the cellular morphology (Figure 2c). Quantitative analysis revealed that the spreading areas of both cell types were approximately 2‐fold greater on stiff gels compared to soft gels (Figure 2d).

We further examined the impact of matrix stiffness on the odontogenic differentiation of hDPSCs and ChDPSCs. Following 7 days of odontogenic induction, both cell types exhibited enhanced differentiation on stiff gels, as evidenced by the higher expression of the differentiation markers DSPP and DMP‐1 (Figure 2e–g). These findings indicate that matrix stiffness is a key regulator of odontogenic differentiation in both hDPSCs and ChDPSCs.

Strikingly, the ITGB1 expression patterns in hDPSCs cultured on soft and stiff gels closely mirrored those observed in ChDPSCs and hDPSCs on standard petri dishes (Figure S8), supporting the validity of our hydrogel system as an in vitro model for stiffness‐related studies. Overall, these findings suggest that the pathological matrix softening is a previously underappreciated contributor to the failed dentin repair observed in teeth with deep caries. This is because matrix stiffness is a critical determinant of the odontogenic differentiation capacity of hDPSCs and ChDPSCs.

### 3.3 Stiff Matrix Promotes the Odontogenic Differentiation of hDPSCs by Enhancing Exosome Release

Exosomes are recognized as essential extracellular vesicles involved in regulating cellular differentiation [30]. However, whether matrix stiffness regulates odontogenic differentiation of hDPSCs through exosomes remains unclear, we therefore hypothesized that exosomes mediate the effects of stiffness on cell differentiation. First, to verify the critical roles of exosomes in the odontogenic differentiation of DPSCs, we employed GW4869, a well‐characterized inhibitor of exosome biogenesis, to suppress exosome secretion during the differentiation process [31]. As illustrated in Figure S9, GW4869‐treated hDPSCs exhibited significantly reduced odontogenic potential, as evidenced by decreased mineral deposition (as assessed by ALP and Alizarin Red S staining) and downregulated expression of the key odontoblast markers DSPP and DMP‐1.

**FIGURE 3:**
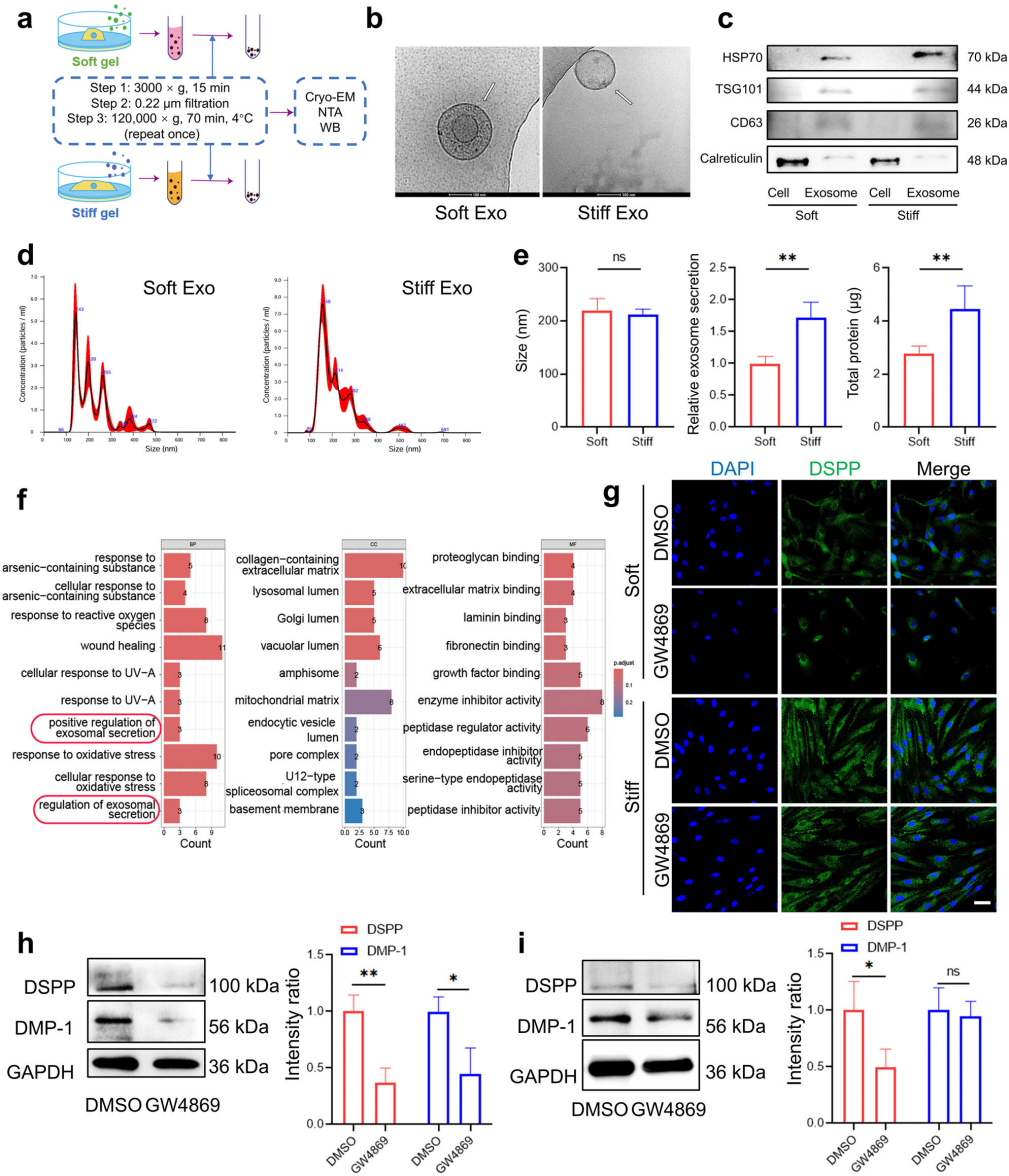
Stiff matrix promotes the odontogenic differentiation of DPSCs through enhancing exosome release. (a) Schematic of exosome isolation from hDPSCs grown on gels with different stiffness. Exosomes were extracted by ultracentrifugation and filtration, and subsequently identified by cryo‐electron microscopy (Cryo‐EM), nanoparticle tracking analysis (NTA), and western blotting (WB). (b) Representative Cryo‐EM images of exosomes secreted by hDPSCs cultured on soft and stiff gels. Scale bar = 100 µm. (c) Western blotting analysis of representative proteins of hDPSCs and exosomes. (d) Nanoparticle tracking analysis (NTA) was employed to characterize the size distribution and concentration of exosomes from hDPSCs on soft and stiff gels (*n* = 3). (e) Comparation of the size, the number of exosomes and the amount of exosomal proteins from hDPSCs cultured on soft and stiff gels (*n* ≥ 3). (f) The diagram of 10 most enriched GO terms, including those related to BP, CC and MF in exosomal proteins from hDPSCs incubated on soft and stiff substrates. (g) Representative confocal images of cells labeled with DSPP (green) and DAPI (blue) for nuclei. Scale bar = 50 µm. (h) The protein levels of DSPP and DMP‐1 in hDPSCs cultured on soft gels with or without 20 µM GW4869 were analyzed by western blotting (*n* = 3). (i) The protein levels of DSPP and DMP‐1 in hDPSCs cultured on stiff gels with or without 20 µM GW4869 were analyzed by western blotting (*n* = 3). Data are expressed as mean ± SD. Statistical analysis was performed using Student's two‐tailed *t*‐test between two groups. *p < 0.05, **p < 0.01, ***p < 0.001, ns not significant.

Next, we investigated the role of exosomes in mediating the effects of matrix stiffness on the odontogenic differentiation of hDPSCs. As shown in Figure S5, stiffness‐related genes correlated strongly with exosome‐related genes, suggesting a potential association between exosomes and stiffness‐regulated differentiation. To explore the effects of stiffness on exosomes, we isolated exosomes secreted from hDPSCs grown on soft and stiff gels (Figure 3a). Exosomes from hDPSCs in both groups exhibited a typical morphology, appearing as circular vesicles with diameters ranging from 50 to 150 nm, consistent with the canonical exosome characteristics (Figure 3b). Western blotting confirmed the enrichment of exosome protein markers (HSP70, TSG101 and CD63) and the depletion of Calreticulin in exosomes compared with parent cells cultured on soft and stiff hydrogels (Figure 3c). Nanoparticle tracking analysis (NTA) revealed that the particle sizes of exosomes in both groups exhibited a similar size distribution, with the majority of particles ranging from 50 to 150 nm (Figure 3d). The presence of particles larger than 150 nm likely resulted from particle aggregation rather than contamination by larger vesicles. We acknowledge that the combination of centrifugation and filtration may not completely eliminate microvesicles smaller than 200 nm. However, given the strong enrichment of exosomal markers and the substantial depletion of calreticulin shown in Figure 3c, any residual non‐exosomal contaminants are unlikely to substantially affect the interpretation of our results.

Interestingly, exosomes from the stiff group showed significantly higher concentrations and total protein content, compared to those from the soft group (Figure 3e). Moreover, GO analyses based on the proteomics indicated that proteins enriched in exosomes from stiff gels are predicted to positively regulate exosome secretion, suggesting a potential positive feedback loop (Figure 3f). Taken together, these findings highlight the influence of matrix stiffness on exosome biogenesis, especially the process of release.

To further verify the roles of matrix stiffness‐associated exosomes in odontogenic differentiation, we investigated the influence of stiffness‐tuned exosomes on the differentiation potential of hDPSCs. hDPSCs cultured on both stiff and soft gels exhibited similar differentiation capacity following GW4869 treatment (Figure 3g), suggesting that the inhibition of exosomes abolished the stiffness‐dependent enhancement of odontogenic differentiation in hDPSCs. Western blot analysis further confirmed that GW4869 significantly impaired expression of odontogenic markers in hDPSCs on both soft and stiff gels (Figure 3h,i). These findings strongly suggest that the stiffness‐mediated enhancement of odontogenic differentiation in hDPSCs is dependent on the increased exosome production.

### 3.4 Matrix Stiffness Regulates Membrane Curvature via Baiap2 and Affects Exosome Release

**FIGURE 4:**
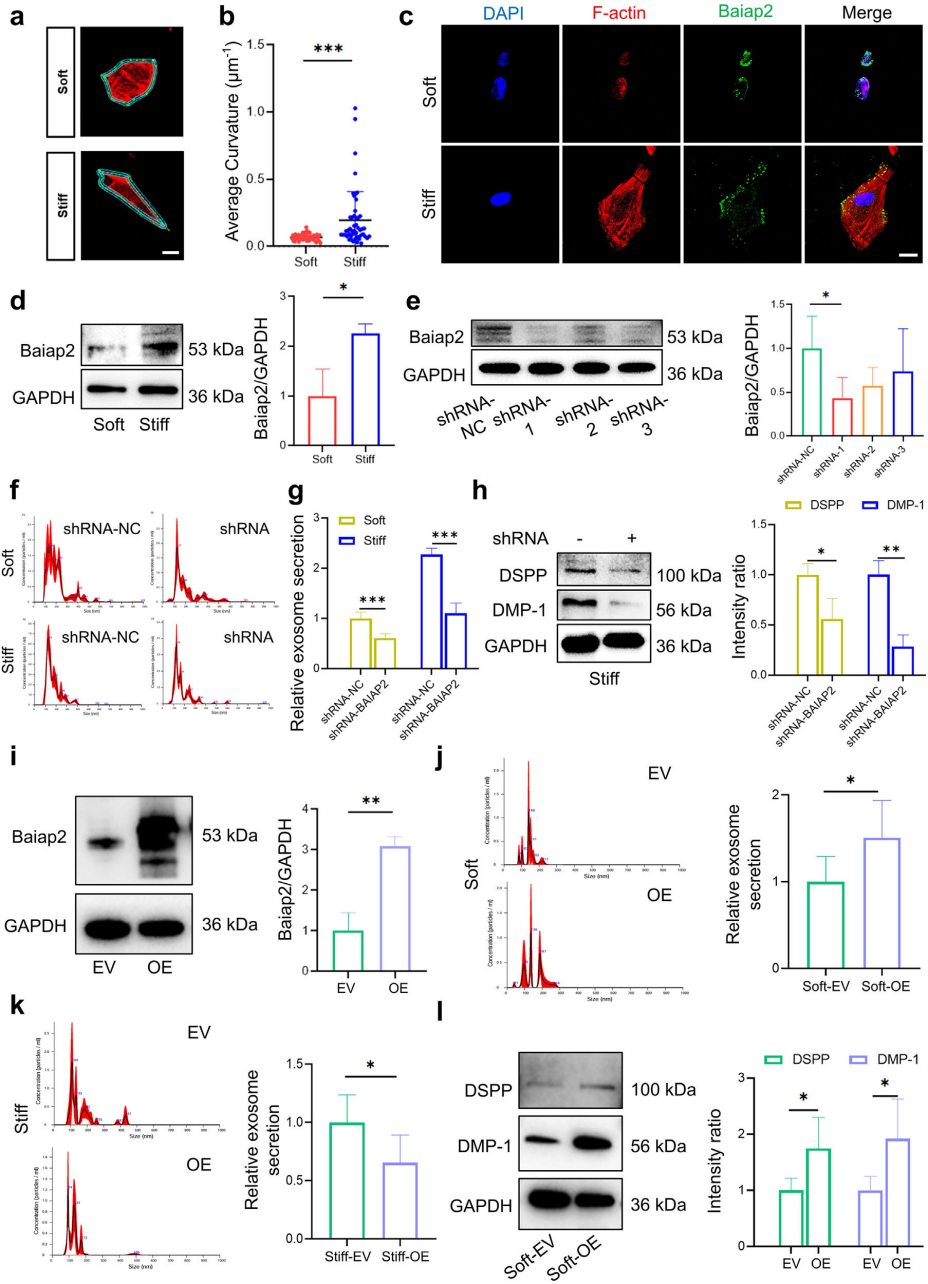
Matrix stiffness regulates membrane curvature via Baiap2, affecting exosome release of DPSCs. (a) Representative images of hDPSCs cultured on soft and stiff gels. Scale bar = 20 µm. (b) Quantification of the average membrane curvature of hDPSCs on substrates with different stiffness (*n* = 50). (c) Representative confocal images of cells labeled with Baiap2 (green), F‐actin (red) and DAPI (blue) for nuclei. Scale bar = 10 µm. (d) The protein levels of Baiap2 in hDPSCs cultured on soft and stiff gels (*n* = 3). (e) The protein levels of Baiap2 after the knockdown by shRNA (*n* = 4). (f) NTA was employed to characterize the size distribution and concentration of exosomes from hDPSCs on soft and stiff gels after the knockdown of Baiap2. (g) Quantification of the exosome release from hDPSCs on soft and stiff gels after the knockdown of Baiap2 (*n* = 6). (h) The protein levels of DSPP and DMP‐1 in hDPSCs cultured on stiff gels with or without knockdown of Baiap2 were analyzed by western blotting (*n* = 3). (i) The protein levels of Baiap2 after the overexpression by shRNA (*n* = 3). EV, empty vector; OE, overexpression of Baiap2. (j) NTA and quantification of the exosome release from hDPSCs on soft gels after the overexpression of Baiap2 (*n* = 6). (k) NTA and quantification of the exosome release from hDPSCs on stiff gels after the overexpression of Baiap2 (*n* = 6). (l) The protein levels of DSPP and DMP‐1 in hDPSCs cultured on soft gels with or without overexpression of Baiap2 were analyzed by western blotting (*n* = 4). Data are expressed as mean ± SD. Statistical analysis was performed using Student's two‐tailed *t*‐test between two groups and one‐way ANOVA followed by Tukey's post‐hoc test among four groups. *p < 0.05, **p < 0.01, ***p < 0.001.

Matrix stiffness has been indicated to enhance odontogenic differentiation partly through the promotion of exosome secretion. However, the precise mechanism linking stiffness to exosome release remain unclear. Membrane curvature, a key indicator of cellular morphology and membrane tension, directly influences vesicle release [32, 33, 34]. Given its fundamental involvement in membrane remodeling and vesicle release, membrane curvature emerges as a key mechanism worthy of focused investigation. Therefore, we hypothesized that membrane curvature is an important mechanism through which matrix stiffness regulates exosome‐mediated differentiation of hDPSCs. To test the role of membrane curvature in exosome release regulated by stiffness, we first measured the average membrane curvature of hDPSCs on soft and stiff gels. The average membrane curvature of hDPSCs on stiff gels was considerably higher, as shown in Figure 4a,b. Furthermore, as evidenced by immunofluorescence images in Figure 4c, Baiap2, a protein that selectively binds to negatively curved membranes and facilitates membrane deformation, was markedly enriched in high‐curvature membrane regions of hDPSCs cultured on stiff gels, whereas it displayed a relatively homogeneous distribution along the inner membrane interface in cells grown on soft gels. There was also a higher expression of Baiap2 in cells on stiffer substrates (Figure 4d). To explore the clinical relevance between biomechanical properties and deep caries, the expression of Baiap2 in hDPSCs and ChDPSCs was also compared (Figure S10). The level of Baiap2 in hDPSCs was higher than that in ChDPSCs, indicating the importance of membrane curvature in caries pathogenesis.

To elucidate the role of Baiap2 in regulating the release of exosomes, we further knocked down Baiap2 in hDPSCs (Figure S11). The results of western blotting in Figure 4e confirmed a significant decrease in Baiap2. The knockdown of Baiap2 diminished the stiffness‐dependent membrane deformation (Figure S12), and reduced exosome production by approximately 50%, a reduction that was observed on both soft and rigid substrates (Figure 4f,g).

To further explore the role of Baiap2 in odontogenic differentiation of hDPSCs, we compared the differentiation capacity of control versus Baiap2‐knockdown hDPSCs cultured on substrates with different stiffness. Our results demonstrated that Baiap2 knockdown significantly inhibited odontogenic differentiation of hDPSCs on stiff substrates, while exerting negligible effects on hDPSCs cultured on soft substrates (Figure 4h, Figures S13 and S14).

To further validate the functional role of Baiap2, we conducted Baiap2 overexpression studies (Figure 4i and Figure S15). On soft gels, Baiap2 overexpression induced a morphological change of hDPSCs from short spindle‐shaped to elongated spindle‐shaped cells (Figures S16 and S17). Moreover, it significantly increased exosome release and promoted odontogenic differentiation of hDPSCs on soft gels (Figure 4j,l and Figure S18). On stiff gels, Baiap2 overexpression markedly reduced both exosome production and odontogenic differentiation of hDPSCs (Figure 4k, Figures S18 and S19). Collectively, these results revealed that Baiap2 is essential for the regulation of exosome release by matrix stiffness, which also play a role in regulating odontogenic differentiation of hDPSCs.

### 3.5 Baiap2 Promotes the Release of Stiffness‐tuned Exosomes Through the Activation of PI3K‐AKT Pathway

**FIGURE 5:**
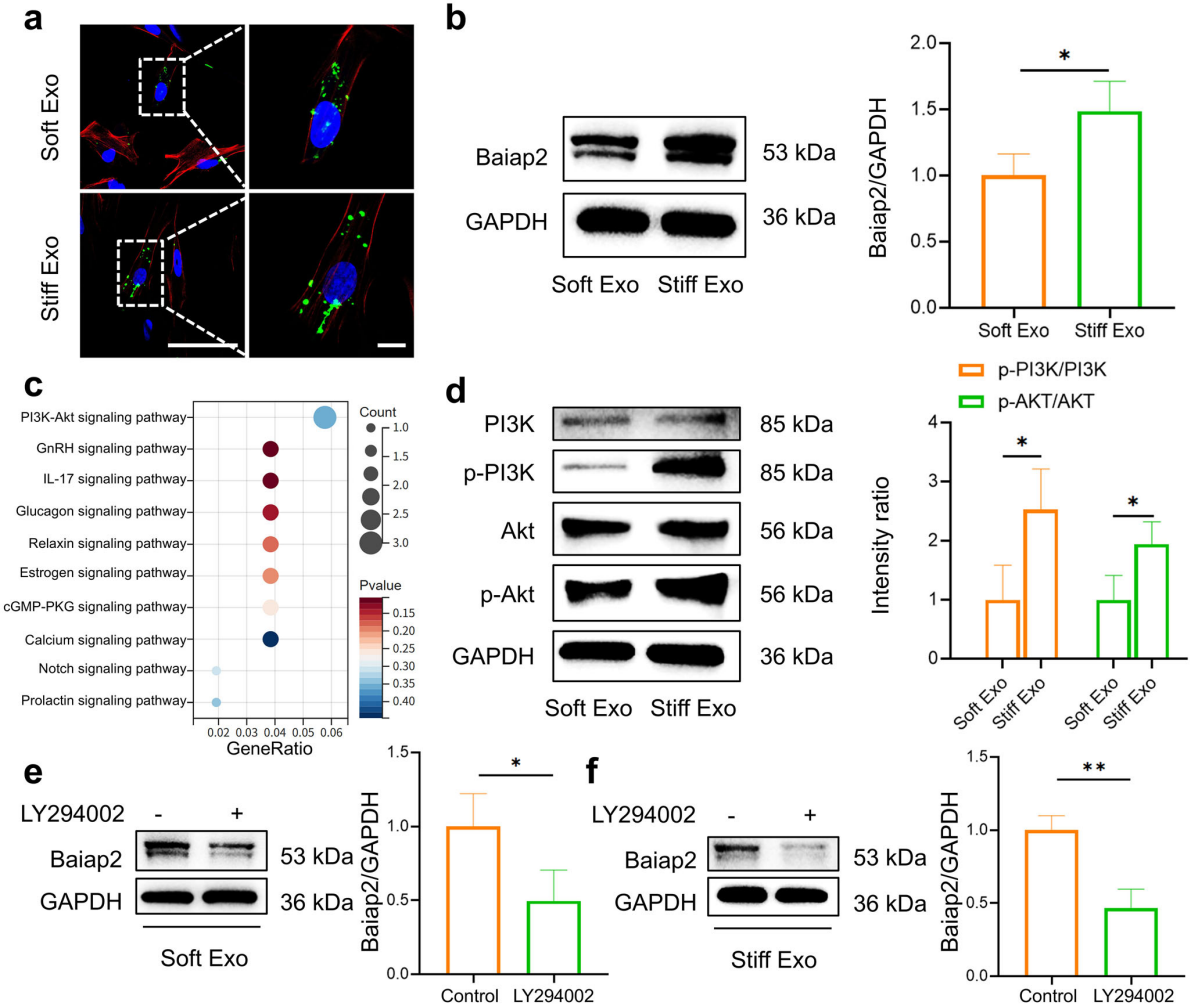
The activation of PI3K‐AKT pathway contributes to the release of stiffness‐tuned exosomes via the increase of Baiap2. (a) Internalization of exosomes (Soft Exo and Stiff Exo) in hDPSCs detected by confocal microscopy. Scale bar = 10 µm. (b) The protein level of Baiap2 in hDPSCs co‐cultured with Soft Exo or Stiff Exo (*n* = 3). (c) The KEGG pathway analysis of hub targets in exosomes from hDPSCs on soft or stiff gels. (d) The activation of PI3K‐AKT in hDPSCs treated with Soft Exo or Stiff Exo at protein level (*n* = 3). (e) The protein level of Baiap2 in hDPSCs treated with Soft Exo with or without LY294002 (*n* = 3). (f) The protein level of Baiap2 in hDPSCs treated with Stiff Exo with or without LY294002 (*n* = 3). Data are expressed as mean ± SD. Statistical analysis was performed using Student's two‐tailed *t*‐test between two groups. *p < 0.05, **p < 0.01, ***p < 0.001.

Based on our previous findings that matrix stiffness enhances Baiap2 expression and exosome secretion, we next investigated the impact of stiffness‐modulated exosomes on recipient hDPSCs. The results in Figure S20 indicate that exosomes are required for cells to sense the matrix stiffness and alter membrane curvature. Using a co‐culture system, we compared the effects of exosomes derived from cells cultured on soft (Soft Exo) versus stiff substrates (Stiff Exo). Confocal microscopy images revealed that Soft Exo and Stiff Exo were internalized by hDPSCs and predominantly accumulated in perinuclear regions, indicating a high uptake efficiency in both groups (Figure 5a). In hDPSCs treated with Stiff Exo, Baiap2 protein expression was substantially elevated versus the Soft Exo group (Figure 5b). The results of KEGG signaling pathway showed that PI3K‐AKT would be the primary mechanism to regulate Baiap2 (Figure 5c). This prediction was then confirmed by an increase in the phosphorylation of key components of PI3K‐AKT pathway in hDPSCs treated with Stiff Exo, as shown in Figure 5d.

To further clarify the upstream mechanism by which stiffness‐tuned exosomes active Baiap2 expression, we treated hDPSCs with the PI3K‐AKT pathway inhibitor LY294002 (Figure S21). Following this inhibition, the expression of Baiap2 in both groups was significantly suppressed in both groups, confirming the critical role of the PI3K‐AKT pathway in regulating Baiap2 (Figure 5e,f). Taken together, these findings suggest that higher matrix stiffness promotes the release of exosomes, with elevated Baiap2 activity to facilitate odontogenic differentiation of hDPSCs. Moreover, stiffness‐modulated exosomes activate the PI3K‐AKT signaling pathway in recipient cells, thereby enhancing Baiap2 expression. This mechanosensitive feedback loop amplifies exosome secretion and establishes a self‐reinforcing cycle that further potentiates odontogenic differentiation.

### 3.6 Kinesin‐1 Modulates the Intracellular Transport and the Release of Stiffness‐Tuned Exosomes

**FIGURE 6:**
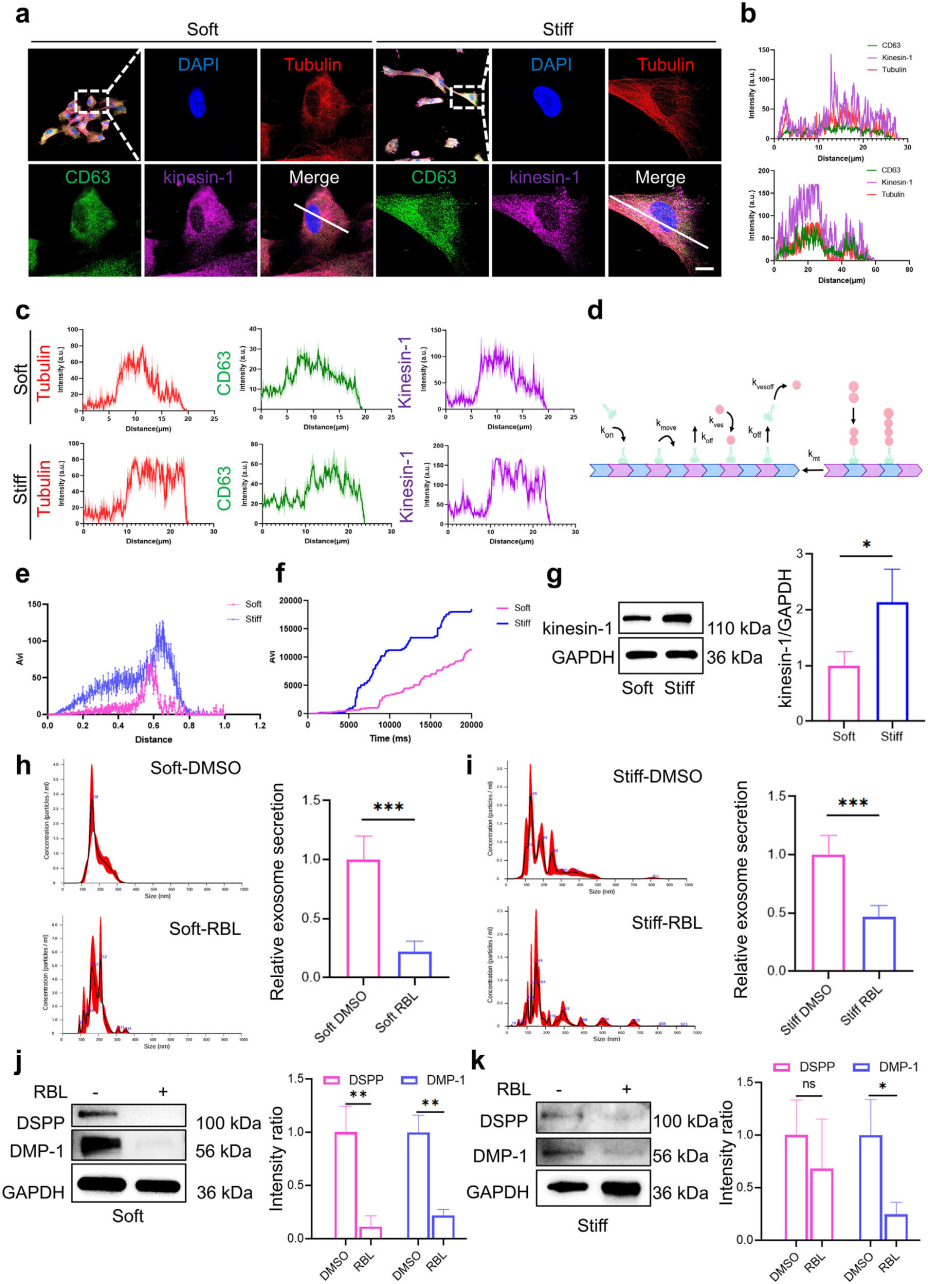
Kinesin‐1 is responsible for regulating the intracellular transport and the release of stiffness‐tuned exosomes. (a) Representative confocal images of hDPSCs labeled with tubulin (red), CD63 (green), kinesin‐1 (purple) and DAPI (blue) for nuclei on soft and stiff substrates. Scale bar = 10 µm. (b) Colocalization analysis of tubulin, CD63 and kinesin‐1. (c) The distribution of tubulin, CD63 and kinesin‐1. The left end of the X‐axis represents the center of the nucleus. (d) Schematic of the current mathematic model. (e) The mathematical model simulation revealed the effects of matrix stiffness on the distribution of MVBs in cells. (f) The mathematical model simulation showed the relationship between matrix stiffness and the release of exosomes. (g) The protein level of kinesin‐1 in hDPSCs on soft and stiff substrates (*n* = 3). (h) NTA and quantification of the exosome release from hDPSCs on soft gels after treatment with kinesin‐1 inhibitor RBL (*n* = 6). (i) NTA and quantification of the exosome release from hDPSCs on stiff gels after treatment with kinesin‐1 inhibitor RBL (*n* = 6). (j) The protein levels of DSPP and DMP‐1 in hDPSCs cultured on soft gels with or without RBL were analyzed by western blotting (*n* = 3). (k) The protein levels of DSPP and DMP‐1 in hDPSCs cultured on stiff gels with or without RBL were analyzed by western blotting (*n* = 3). Data are expressed as mean ± SD (mean ± SEM for Figure 6c). Statistical analysis was performed using Student's two‐tailed *t*‐test between two groups. *p < 0.05, **p < 0.01, ***p < 0.001, ns not significant.

Although our study demonstrated that in hDPSCs cultured on stiff gels, Baiap2 interacts with the PI3K‐AKT pathway to promote exosome secretion and thereby enhance odontogenic differentiation, its effects appear limited under soft substrate conditions, which mimic the microenvironment of deep carious pulp. From a mechanistic standpoint, this suggests that simply increasing Baiap2 may not be sufficient to promote odontogenic differentiation in a soft matrix environment. Given that kinesins drive the transport of vesicles to the cell periphery and Baiap2 regulates the positioning of vesicles near the membrane, they might act in complementary pathways to regulate exosomes [35]. As kinesin‐1 is a crucial motor protein influencing the intracellular trafficking state of exosomes before their release, we next investigated whether kinesin‐1 serves as a mechano‐transducer in this process and represent a potential mechanistic target for further investigation [36]. As shown in Figure 6a,b, the transport and release of exosomes in hDPSCs are indeed dependent on kinesin‐1. hDPSCs on stiff gels exhibited a greater number of MVBs with higher levels of kinesin‐1, and the distribution was localized closer to the cell membrane (Figure 6c,g). Furthermore, based on the observed variations in membrane curvature under different matrix stiffness, we developed a mathematical model to describe the interactions between kinesin‐1 and microtubule to predict the distribution of MVBs or the release of exosomes in hDPSCs (Figure 6d–f). The simulation results were highly consistent with our experimental findings described above.

**FIGURE 7:**
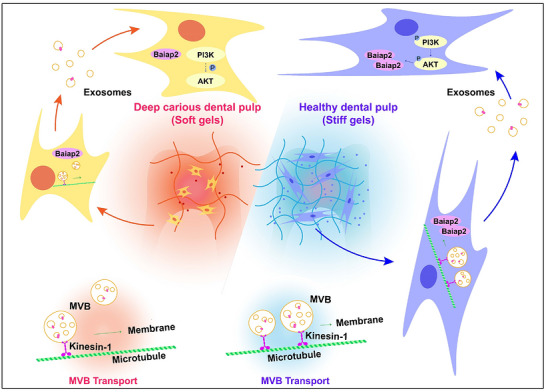
Graphic abstract for the current study.

To further validate the role of kinesin‐1 in the transport of MVBs and release of exosomes, hDPSCs on both soft and stiff gels were treated with a kinesin‐1 inhibitor rose bengal lactone (RBL) (Figure S22), and the changes in exosome production were assessed. As shown in Figure 6h,i, the secretion of exosomes from hDPSCs on both soft and stiff gels was significantly decreased. More importantly, inhibition of kinesin‐1 by RBL markedly suppressed the odontogenic differentiation of hDPSCs on both soft and stiff gels, an effect that could not be reproduced by Baiap2 (Figure 6j,k, Figure S23). These findings indicate that kinesin‐1 may serve as a pivotal regulator of MVB transport and exosome release during the process of hDPSCs sensing matrix stiffness. Thus, kinesin‐1 is not only a motor protein, but also a potential target to enhance odontogenic differentiation of hDPSCs in deep caries (Figure 7).

## 4 Discussions

Deep caries creates a pathological microenvironment characterized by persistent bacterial invasion, chronic inflammation and the upregulation of MMPs, which degrade the ECM and impair tissue regeneration [37, 38]. Such pathological matrix remodeling is intrinsically associated with a reduction in matrix stiffness, a biophysical cue which has been increasingly recognized as a pivotal regulator of stem cell fate [39]. However, the impact of stiffness on the odontogenic potentials of hDPSCs has remained poorly understood. Here, we report a significant reduction in matrix stiffness in deep carious pulp and elucidate a previously unrecognized mechanism whereby stiffness regulates the odontogenic differentiation of hDPSCs. Specifically, this study shows that increased matrix stiffness augments exosome release by regulating kinesin‐1 and Baiap2‐PI3K‐AKT loop, thereby activating the odontogenic differentiation of hDPSCs.

The pathogenesis of deep caries has long been scrutinized through a biochemical perspective. Compelling studies have established that inflammation and bacterial invasion associated with deep caries would impair hDPSCs, thereby limiting the reparative capacity of dental pulp [40, 41]. However, the dental pulp microenvironment is an integration of biochemical and biophysical cues. Matrix stiffness, which is one of the most extensively studied biophysical cues, has emerged as a potent regulator of stem cell behaviors [42, 43]. In deep caries, acid demineralization and enzymatic collagen breakdown would inevitably alter the matrix stiffness of dental pulp [44]. This led matrix stiffness to be hypothesized as a previously overlooked hallmark of deep caries. Our study demonstrates that the stiffness of dental pulp tissues in deep caries differs significantly from that of healthy pulp tissues, which affects the differentiation fate of hDPSCs. Our work provides experimental evidence to support this hypothesis, elucidating how changes in matrix stiffness of deep carious dental pulp influenced the odontogenic differentiation of hDPSCs. The softened matrix of dental pulp is not merely a passive consequence of deep caries, but actively disrupts the odontogenic differentiation of hDPSCs. By identifying matrix stiffness as a key regulator of hDPSCs in deep caries, our work provides a more holistic understanding of deep caries and highlights the significance of biophysical cues in dental diseases.

Matrix stiffness has been established as a determinant of hDPSC fate in deep caries, but the precise mechanisms transducing this biophysical signal into odontogenic differentiation remained unclear. Many studies have shown that matrix stiffness can guide stem cell differentiation through classical mechanotransduction pathways, particularly integrin‐mediated signals [29, 45, 46]. Consistent with these reports, we observed the differences of ITGB1 in hDPSCs cultured on gels with different stiffness. However, emerging evidence suggests that matrix stiffness can modulate cell behaviors through exosomes [47, 48]. Exosomes have been recognized as essential carriers of proteins, lipids and nucleic acids in intercellular communication [49]. In this study, we further indicate that exosomes serve as a key downstream effector, translating matrix stiffness into odontogenic differentiation of hDPSCs. Our study thus provides a novel mechanism whereby matrix stiffness partially regulates the odontogenic differentiation of hDPSCs via exosome‐mediated signals.

The relationship between matrix stiffness and the secretion of exosomes remains controversial, with reports of positive correlation in cancer cells and chondrocytes [19, 47, 48], but a negative one in stem cells [20]. This discrepancy highlights the complexity of the underlying mechanobiological pathways. Exosomes are membrane‐derived vesicles formed through endosomal trafficking and membrane budding, consequently their biogenesis is sensitive to the biophysical features of the cell membrane [50]. On stiffer substrates, cells exhibit enhanced cytoskeletal contractility, which can be transmitted to the plasma membrane and elevate membrane tension [51]. Increased membrane tension has been reported to facilitate vesicle release, providing a potential mechanical link between matrix stiffness and exosome secretion [52, 53]. Furthermore, given the tight coupling between membrane tension and curvature, we speculated membrane curvature acts as a critical mechanism through which matrix stiffness governs exosome secretion [51, 54, 55].

Membrane curvature proteins containing a Bin/Amphiphysin/Rvs (BAR) domain can modulate the distribution of membrane tension, a process that is, in turn, reciprocally regulated the assembly of BAR proteins [56]. Notably, Baiap2, a representative inverse‐BAR (I‐BAR) domain protein suppresses inflammatory responses in macrophages cultured on stiffer substrates [57]. We therefore hypothesized that Baiap2 mediates the increased exosome secretion from hDPSCs cultured on stiffer substrates. Our findings confirmed this hypothesis, demonstrating that increasing matrix stiffness promotes exosome secretion by hDPSCs via Baiap2, at least within the sub‐1000 Pa range. This established link between membrane curvature and exosome release suggests a novel strategy for enhancing exosome production. Indeed, membrane curvature can function as a potent biomechanical signal within the cellular microenvironment, influencing various cellular behaviors [58]. Consequently, manipulating membrane curvature, for instance, by applying nanoscale surface topographies, may represent a promising strategy to enhance exosome yield and optimize production [59].

However, the mechanism through which Baiap2 exerts its function is complex and governed by a delicate balance between its expression level and the biomechanical properties of the extracellular matrix. Specifically, Baiap2 knockdown significantly inhibited odontogenic differentiation of hDPSCs on stiff gels but had negligible effects on soft gels. This disparity can be attributed to several factors. First, lower stiffness strongly suppresses odontogenic differentiation of hDPSCs, making Baiap2 a less critical factor under this condition. Nevertheless, Baiap2 overexpression partially rescues differentiation, suggesting that Baiap2 can support odontogenic differentiation on soft gels. Secondly, although Baiap2 knockdown reduced exosome quantity, the cargo within exosomes may change, partially compensating for the loss of odontogenic differentiation. Thirdly, the secretion of soluble cytokines might also contribute to differentiation, although this remains speculative without experimental evidence. Collectively, the negligible effect on soft gels likely results from mechanical inhibition and functional compensation. In contrast, Baiap2 overexpression produced opposing effects on soft and stiff gels. On soft gels, Baiap2 overexpression promoted exosome release and then odontogenic differentiation of hDPSCs, which further support the pivotal role of Baiap2. However, the overexpression on stiff gels unexpectedly inhibited these processes. This may be explained by the activation of upstream negative feedback mechanisms through Baiap2 overexpression on stiff gels, which suppress exosome secretion and then impair odontogenic differentiation. Taken together, these results demonstrate that the function of Baiap2 depends on its expression level and the mechanical properties of the extracellular matrix.

While stiffness‐enhanced exosome release has been reported in other cell types, our study uncovers a novel bidirectional feedback loop in hDPSCs. Stiff gels promote exosome secretion via Baiap2, and these exosomes activate PI3K‐AKT pathway to upregulate Baiap2, further enhancing exosome release. This reciprocal mechanism, supported by comprehensive proteomic analysis and biological validation, represents a previously unexplored pathway. Importantly, Baiap2 expression increases with matrix stiffness and is suppressed upon PI3K‐AKT inhibition, establishing it as a downstream effector of stiffness‐induced signaling. Through this effector‐mediated feedback loop, cells amplify their response to matrix stiffness, influencing membrane dynamics and promoting sustained exosome release. Interestingly, such a feedback‐amplified mechanism in cellular responses to stiffness is not limited to exosome biology. In the tumor microenvironment, cancer‐associated fibroblasts (CAFs) and ECM stiffness also form a positive feedback loop. Excessive collagen deposition by activated CAFs increases the stiffness of ECM, which in turn promotes more fibroblasts transform into CAFs [60]. In brief, these findings highlight a shared mechanism in which mechanical cues and intracellular signaling mutually reinforce each other, so that cells can maintain homeostasis and adaptability under varying mechanical conditions.

The release of exosomes relies on the fusion of MVBs with the plasma membrane, a process regulated by kinesin‐driven transport of MVBs along microtubules [61]. To elucidate a more precise regulatory mechanism, we investigated the specific types of kinesins involved in MVB trafficking. Kinesin‐1 has been shown to regulate MVB transport and exosome release, as well as sensing stiffness to influence myogenic differentiation [35, 62]. Using RBL to inhibit kinesin‐1, our findings demonstrate that kinesin‐1 is critical for the transport and release of stiffness‐tuned exosomes, which then modulated the odontogenic differentiation of hDPSCs in a stiffness‐dependent manner, which was unknown before. To quantitatively analyze this process, we developed a mathematical model to describe the dynamic behaviors of MVB transport in response to different stiffness. By incorporating parameters such as kinesin activity and microtubule stability, this model provides a theoretical framework for understanding the biophysical mechanisms underlying MVB trafficking and exosome release [63]. Mechanistically, increased matrix stiffness enhances the binding of kinesin‐1 to microtubules, thereby promoting the directional transport of a greater number of MVBs towards the plasma membrane per unit time and resulting in higher levels of exosome secretion. Consequently, these stiffness‐tuned exosomes act as mediators to regulate odontogenic differentiation of hDPSCs. Our results provide stronger theoretical support for the role of kinesin‐1 in linking matrix stiffness to exosome‐driven differentiation.

Focusing on the relationship between matrix stiffness and deep caries, we have preliminarily elucidated the mechanism by which stiffness influences odontogenic differentiation of hDPSCs through affecting exosomes. Clinically, the reduced stiffness of dental pulp in deep caries may compromise the mechano‐responsive exosomal pathway identified here. This finding provides a mechanistic rationale for future pulp‐capping strategies, suggesting that the capacity of pulp‐capping materials to modulate the stiffness of dental pulp should be considered as a critical factor in material design. Preserving the physiological mechanical microenvironment of pulp may therefore be as important as infection control or biochemical stimulation in achieving optimal reparative outcomes. However, certain limitations should be acknowledged. First, a primary limitation lies in the specific bioactive components within the exosomes that drives the observed PI3K‐AKT activation and subsequent differentiation remain unidentified. Secondly, our investigation focused exclusively on hDPSCs, despite the fact that deep caries involves a dynamic interplay of multiple cell types. Therefore, future work must aim to expand the in vitro system to include other critical cell types. Thirdly, the therapeutic potential of stiffness‐regulated exosomes remains to be fully elucidated. Our current study cannot fully recapitulate the complex deep caries microenvironment in vivo. Therefore, developing a monitorable animal model for dental pulp stiffness is necessary to validate the therapeutic potential of stiffness‐tuned exosomes in vivo. Finally, it is also crucial to consider the role of inflammation and immune responses in deep caries. Further studies investigating how inflammatory signals interact with matrix stiffness in deep caries will be important to fully understand the pathophysiology of this disease.

## 5 Conclusions

Taken together, in this study, we investigated the effects of the impaired extracellular matrix stiffness on DPSCs and underlying roles of stiffness‐tuned exosome trafficking and release in deep caries. Our findings reveal that matrix stiffness can influence the differentiation potential of DPSCs by regulating exosome production, and offer a novel mechanistic insight into the role of the biophysical extracellular microenvironment in deep caries pathology.

## Author Contributions

All authors contributed to the study conception and design. **B.J. S.D**. and **Y.J**. contributed equally to this work. Conceptualization: **B.J**., **S.D**., and **L.N**.; Methodology: **B.J**., **S.D**., **Y.J**., **Z.X**., **P.L**. and **J.H**.; Formal Analysis: **Z.D**., **Y.L**., and **R.Z**.; Investigation: **B.J**., **S.D**., **Y.J**., and **Z.D**.; Resources: **J.H**., **B.C**., and **L.N**.; Writing – Original Draft: **B.J**. and **S.D**.; Writing – Review & Editing: All authors; Supervision: **J.H**., **B.C**., and **L.N**.; Funding Acquisition: **L.N**. All authors contributed to the study conception and design.

## Ethics Approvals

This study involves human participants and was approved by the ethics committee of Xi'an Jiaotong University (KY‐GXB‐20230002). Participants gave informed consent to participate in the study before taking part. All samples were anonymized before use in the experiments.

## Conflicts of Interest

The authors declare no conflicts of interest.

## Supporting information




**Supporting File 1**: advs76908‐sup‐0001‐SuppMat.docx.

## Data Availability

The data that support the findings of this study are available from the corresponding author upon reasonable request.
