# Supplementary material for: Matrix Stiffness Enhances Odontogenic Differentiation of DPSCs through Membrane Curvature Protein Baiap2‐Modulated Exosome Release
Source: Adv Sci (Weinh). 2026 Aug 11;13(60):e76908. doi: 10.1002/advs.76908 (PMC13460413; doi:10.1002/advs.76908)
Supplement: Supplementary file 1 [file ADVS-13-e76908-s001.docx]

**Supplementary Materials**

**Figure Legends**

**Figure S1.** The culture and identification of hDPSCs and ChDPSCs.

**Figure S2.** hDPSCs and ChDPSCs proliferation determined by EdU assays.

**Figure S3.** Heatmap of dental pulp donor characteristics.

**Figure S4.** Histological sections of sound dental pulp and deep carious pulp with hematoxylin/eosin.

**Figure S5.** Single-cell RNA sequencing analysis of sound and deep carious dental pulp tissues.

**Figure S6.** Swelling behavior of hydrogels with different stiffness at indicated time points.

**Figure S7.** The morphology and viability of hDPSCs and ChDPSCs on the soft and stiff gels.

**Figure S8.** The expression of Integerin β1 in hDPSCs grown on soft/stiff gels.

**Figure S9.** Exosomes are necessary for the odontogenic differentiation of hDPSCs.

**Figure S10.** Western blot analysis of Baiap2 expression in hDPSCs and ChDPSCs.

**Figure S11.** Representative images of hDPSCs infected by the lentivirus stably.

**Figure S12.** Representative confocal images of hDPSCs grown on soft and stiff gels with or without the knockdown of Baiap2.

**Figure S13.** Representative confocal images for DSPP in hDPSCs grown on soft and stiff gels with or without the knockdown of Baiap2.

**Figure S14.** The protein levels of DSPP and DMP-1 in hDPSCs cultured on soft gels with or without knockdown of Baiap2

**Figure S15.** Representative images of hDPSCs stably infected by the lentivirus for Baiap2 overexpression.

**Figure S16.** Representative confocal images of hDPSCs grown on soft and stiff gels stably infected by the lentivirus for Baiap2 overexpression.

**Figure S17.** Representative confocal images of hDPSCs grown on soft and stiff gels with or without the overexpression of Baiap2.

**Figure S18.** Representative confocal images for DSPP in hDPSCs grown on soft and stiff gels with or without the overexpression of Baiap2.

**Figure S19.** The protein levels of DSPP and DMP-1 in hDPSCs cultured on stiff gels with or without overexpression of Baiap2.

**Figure S20.** The regulation of membrane curvature requires exosomes.

**Figure S21.** LY294002 inhibits PI3K-AKT pathway in hDPSCs incubated with stiffness-tuned exosomes.

**Figure S22.** The expression and the activity of kinesin-1 could be efficiently inhibited by RBL.

**Figure S23.** Representative images of DSPP staining in hDPSCs grown on soft and stiff gels with or without 40 μM RBL.

**Fig S1.** The culture and identification of hDPSCs and ChDPSCs. (a) Morphology, adipogenic and osteogenic differentiation of hDPSCs. Scale bar = 100 μm. Surface expression of positive (CD73, CD90, and CD105) and negative (CD45) markers on hDPSCs were evaluated using flow cytometry (*n* = 3). (b) The similar identification of ChDPSCs through morphology, multi-differentiation, and flow cytometric analysis (*n* = 3).

**Fig S2.** hDPSCs and ChDPSCs proliferation determined by EdU assays (*n* = 6). Scale bar = 50 μm. Data are expressed as mean ± SD. Statistical analysis was performed using Student’s two-tailed *t*-test between two groups. **P* < 0.05, ***P* < 0.01, ****P* < 0.001, ns not significant.

**Fig S3.** Heatmap of dental pulp donor characteristics.

**Fig S4.** Histological sections of sound dental pulp and deep carious pulp with hematoxylin/eosin. Scale bar = 100 μm (*n* = 5).

**Fig S5.** Single-cell RNA sequencing analysis of sound and deep carious dental pulp tissues. (A) The t-SNE plot and violin plot of the stem cell populations. Violin plot demonstrating the expression levels of selected MSC-related genes MCAM, THY1 and PTPRC. (B) The heatmap of the correlation figure for genes related to exosomes, membrane curvature and integrin family.

**Fig S6.** Swelling behavior of hydrogels with different stiffness at indicated time points (*n* = 4). Data are expressed as mean ± SD.

**Fig S7.** The morphology and viability of hDPSCs and ChDPSCs on the soft and stiff gels (*n* = 4). Scale bar = 50 μm. The number of viable cells were monitored in day 1 and day 3. Data are expressed as mean ± SD. Statistical analysis was performed using Student’s two-tailed *t*-test between two groups. **P* < 0.05, ***P* < 0.01, ****P* < 0.001, ns not significant.

**Fig S8.** The expression of ITGB1 in hDPSCs grown on soft/stiff gels. (a) Western blot analysis of ITGB1 expression in hDPSCs obtained from soft and stiff gels (*n* = 4). (b) Representative confocal images of ITGB1 staining in hDPSCs grown on soft and stiff gels. Scale bar = 10 μm. Data are expressed as mean ± SD. Statistical analysis was performed using Student’s two-tailed *t*-test between two groups. **P* < 0.05, ***P* < 0.01, ****P* < 0.001, ns not significant.

**Fig S9.** Exosomes are necessary for the odontogenic differentiation of hDPSCs. (a) ALP activities were assessed to identify the role of exosomes in the odontogenic differentiation of hDPSCs (*n* = 3). (b) Alizarin red staining and quantitative analysis of mineralized nodule formation were used to characterize the mineralized nodules (*n* = 4). Scale bar = 100 μm. (c) Western blot analysis of DSPP and DMP-1 expression in hDPSCs treated with DMSO or 20 μM GW4869 (*n* = 3). Data are expressed as mean ± SD. Statistical analysis was performed using Student’s two-tailed *t*-test between two groups. **P* < 0.05, ***P* < 0.01, ****P* < 0.001, ns not significant.

**Fig S10.** Western blot analysis of Baiap2 expression in hDPSCs and ChDPSCs (*n* = 4). Data are expressed as mean ± SD. Statistical analysis was performed using Student’s two-tailed *t*-test between two groups. **P* < 0.05, ***P* < 0.01, ****P* < 0.001, ns not significant.

**Fig S11.** Representative images of hDPSCs infected by the lentivirus stably. Scale bar = 50 μm.

**Fig S12.** Representative confocal images of hDPSCs grown on soft and stiff gels with or without the knockdown of Baiap2. Cells were labeled with Baiap2 (yellow), F-actin (red) and DAPI (blue) for nuclei. Scale bar = 20 μm.

**Fig S13.** Representative confocal images for DSPP in hDPSCs grown on soft and stiff gels with or without the knockdown of Baiap2. Cells were labeled with DSPP (yellow) and DAPI (blue) for nuclei. Scale bar = 20 μm.

**Fig S14.** The protein levels of DSPP and DMP-1 in hDPSCs cultured on soft gels with or without knockdown of Baiap2 (*n* = 3). Data are expressed as mean ± SD. Statistical analysis was performed using Student’s two-tailed *t*-test between two groups. **P* < 0.05, ***P* < 0.01, ****P* < 0.001, ns not significant.

**Fig S15.** Representative images of hDPSCs stably infected by the lentivirus for Baiap2 overexpression. Scale bar = 50 μm.

**Fig S16.** Representative confocal images of hDPSCs grown on soft and stiff gels stably infected by the lentivirus for Baiap2 overexpression. Scale bar = 50 μm.

**Fig S17.** Representative confocal images of hDPSCs grown on soft and stiff gels with or without the overexpression of Baiap2. Cells were labeled with Baiap2 (yellow), F-actin (purple) and DAPI (blue) for nuclei. Scale bar = 20 μm.

**Fig S18.** Representative confocal images for DSPP in hDPSCs grown on soft and stiff gels with or without the overexpression of Baiap2. Cells were labeled with DSPP (yellow) and DAPI (blue) for nuclei. Scale bar = 50 μm.

**Fig. S19.** The protein levels of DSPP and DMP-1 in hDPSCs cultured on stiff gels with or without overexpression of Baiap2 (*n* = 5). Data are expressed as mean ± SD. Statistical analysis was performed using Student’s two-tailed *t*-test between two groups. **P* < 0.05, ***P* < 0.01, ****P* < 0.001, ns not significant.

**Fig S20.** The regulation of membrane curvature requires exosomes. (a) Representative confocal images of the morphology of cells grown on soft and stiff gels with or without DMSO/20 μM GW4869. Scale bar = 20 μm. (b) Quantitative analysis of average curvature of cells in each group (*n* ≥ 20). Data are expressed as mean ± SD. Statistical analysis was performed using one-way ANOVA followed by Tukey’s post-hoc test among six groups. **P* < 0.05, ***P* < 0.01, ****P* < 0.001, ns not significant.

**Fig S21.** LY294002 inhibits PI3K-AKT pathway in hDPSCs incubated with stiffness-tuned exosomes. (a) Western blot analysis of PI3K, p-PI3K, AKT, p-AKT expression in hDPSCs treated with 50 μg/mL exosomes from cells grown on softer gels (*n* = 3). (b) Similar analysis in hDPSCs treated with 50 μg/mL exosomes from cells grown on stiffer gels (*n* = 3). Data are expressed as mean ± SD. Statistical analysis was performed using Student’s two-tailed *t*-test between two groups. **P* < 0.05, ***P* < 0.01, ****P* < 0.001, ns not significant.

**Fig S22.** The expression and the activity of kinesin-1 could be efficiently inhibited by RBL. (A) Representative images and colocalization analysis of CD63, kinesin-1 and tubulin in hDPSCs on soft and stiff gels with or without 40 μM RBL. Scale bar = 10 μm. (B) Western blot analysis of kinesin-1 expression in hDPSCs on soft and stiff gels treated with DMSO or 40 μM RBL (*n* = 3). Data are expressed as mean ± SD. Statistical analysis was performed using Student’s two-tailed *t*-test between two groups. **P* < 0.05, ***P* < 0.01, ****P* < 0.001, ns not significant.

**Fig S23.** Representative images of DSPP staining in hDPSCs grown on soft and stiff gels with or without 40 μM RBL. Scale bar = 50 μm.

**Supplemental Table 1.** List of antibodies used for flow cytometry.

| **Primary Antibody** | **Dosage/test** | | **Source** |
| --- | --- | --- | --- |
| FITC Anti-Human CD45 Antibody (E-AB-F1137C) | 5 μL | Elabscience | |
| FITC Anti-Human CD73 Antibody (E-AB-F1242C) | 5 μL | Elabscience | |
| FITC Anti-Human CD90 Antibody (E-AB-F1167C) | 5 μL | Elabscience | |
| PE Anti-Human CD105 Antibody (E-AB-F1310D) | 5 μL | Elabscience | |
| FITC Anti-Human CD29 Antibody (E-AB-F1049C) | 5 μL | Elabscience | |

**Supplemental Table 2.** List of antibodies used for western blotting.

| **Primary Antibody** | **Dilution** | **Source** |
| --- | --- | --- |
| Rabbit anti-GAPDH (10494-1-AP) | 1:20000 | Proteintech |
| Rabbit anti-ITGB1 (12594-1-AP) | 1:1000 | Proteintech |
| Mouse anti-DSPP (sc-73632) | 1:500 | Santa Cruz Biotechnology |
| Rabbit anti-DMP-1 (YN0112) | 1:500 | Immunoway |
| Rabbit anti-HSP70 (YT5424) | 1:1000 | Immunoway |
| Rabbit anti-TSG101 (YM8150) | 1:500 | Immunoway |
| Mouse anti-CD63 (YM4918) | 1:500 | Immunoway |
| Rabbit anti-Calreticulin (YM8162) | 1:500 | Immunoway |
| Rabbit anti-Baiap2 (A5337) | 1:1000 | ABclonal |
| Rabbit anti-kinesin-1 (AB167429) | 1:2000 | Abcam |
| Rabbit anti-PI3K (T40115) | 1:500 | Abmart |
| Rabbit anti-p-PI3K (T40116) | 1:500 | Abmart |
| Rabbit anti-Akt (T55561) | 1:500 | Abmart |
| Rabbit anti-p-Akt (T40067) | 1:500 | Abmart |

**Supplemental Table 3.** Compositions of PA hydrogels.

| **Component** | **Soft gels (μL)** | **Stiff gels (μL)** | **Manufacturer** |
| --- | --- | --- | --- |

| 50% Acr (w/v) | 240 | 240 | Macklin, China |
| --- | --- | --- | --- |
| 1.25% Bis (w/v) | 50 | 150 | Macklin, China |
| 10% APS (w/v) | 20 | 20 | Macklin, China |
| ddH_2_O | 1688 | 1588 | / |
| TEMED | 2 | 2 | Boster, China |

Abbreviations: w/v, weight/volume.

**Supplemental Table 4.** List of antibodies used for immunofluorescence staining.

| **Primary Antibody** | **Dilution** | **Source** |
| --- | --- | --- |
| Mouse anti-DSPP (sc-73632) | 1:50 | Santa Cruz Biotechnology |
| Rabbit anti-Baiap2 (A5337) | 1:150 | ABclonal |
| Mouse anti-CD63 (YM4918) | 1:50 | Immunoway |
| Rabbit anti-kinesin-1 (AB167429) | 1:200 | Abcam |
| Rabbit anti-ITGB1 (12594-1-AP) | 1:50 | Proteintech |

**Supplemental Table 5.** Demographic and clinical characteristics of dental pulp donors.

| **Group** | **Age (year)** | **Gender** | **Tooth position** | **Matrix stiffness (Pa)** |
| --- | --- | --- | --- | --- |

| Normal | 24 | Male | Third molar | 1289.50 |
| --- | --- | --- | --- | --- |
| Normal | 25 | Female | Third molar | 759.48 |
| Normal | 24 | Female | Third molar | 1290.20 |
| Normal | 25 | Female | Third molar | 800.46 |
| Normal | 19 | Female | Third molar | 974.90 |
| Deep caries | 21 | Male | Third molar | 280.40 |
| Deep caries | 23 | Male | Third molar | 296.83 |
| Deep caries | 25 | Male | Third molar | 438.75 |
| Deep caries | 18 | Female | Third molar | 392.62 |
| Deep caries | 21 | Female | Third molar | 393.58 |
